# Hyoid bone position with bone-borne or tooth-borne surgically assisted rapid palatal expansion: a retrospective cohort study

**DOI:** 10.1007/s00784-025-06529-5

**Published:** 2025-10-23

**Authors:** Jonas Q. Schmid, Thomas Stamm, Claudius Middelberg, Bernhard Wiechens, Ariane Hohoff, Moritz Kanemeier

**Affiliations:** 1 Department of Orthodontics, University of Münster, Albert-Schweitzer-Campus 1, 48149 Münster, Germany; 2 Department of Orthodontics, University Medical Center Göttingen, Robert-Koch-Str. 40, 37075 Göttingen, Germany

**Keywords:** Surgically assisted rapid palatal expansion, Maxillary expansion, Bone-borne, Tooth-borne, Distraction, Expansion appliance, Orthognathic surgery, Airway, Obstructive sleep apnea

## Abstract

**Objectives:**

There is a lack of evidence whether hyoid bone position changes with surgically assisted rapid palatal expansion (SARPE), which could improve the airway. The aim of this study was to (1) evaluate the differences in hyoid bone position with SARPE, (2) assess whether there are differences between bone-borne or tooth-borne expansion appliances, and (3) investigate possible relationships between expansion values and hyoid bone position.

**Materials and Methods:**

Eligible for inclusion in this retrospective cohort study were adult patients who underwent SARPE using bone-borne or tooth-borne expansion appliances. The Hyrax group (n=32; female/male 20/12; mean age 26.0 ± 9.1 years) was treated with a hyrax appliance and the TPD group (n=33; female/male 15/18; mean age 25.0 ± 8.0 years) was treated with a transpalatal distractor. The position of the hyoid bone was determined on lateral cephalograms before (${t}_{\text{0}}$) and after SARPE (${t}_{\text{1}}$) using eight measurements and five landmarks. Arch width was measured at dental landmarks on STL files and the amount of maxillary expansion was calculated.

**Results:**

Hyoid bone position remained generally stable, with changes not exceeding 2 mm. The only statistically significant change in the overall cohort was observed for the sagittal distance Hy-Me, which decreased from 44.23 mm to 43.40 mm (${p}\, {=0.005}$). Significant differences between groups were noted for the vertical distance Hy-Me’, with the Hyrax group showing a mean change of -1.95 mm (${p}\, {=0.001}$) and the TPD group showing an increase of 0.75 mm (${p}\, {=0.001}$), while the craniocervical inclination $\alpha$ decreased by 1.25$^{\circ }$ in the TPD group (${p}\, {=0.036}$). All other measurements showed no significant changes and possible correlations between the expansion values and the position of the hyoid bone were inconclusive.

**Conclusions:**

While the results indicate small, statistically significant, but probably clinically negligible movements of the hyoid bone after SARPE, the hyoid position remained generally stable and changes were not dependent on the appliance used or the amount of expansion.

**Clinical Relevance:**

Overall, the observed changes were not substantial enough to support any clinically meaningful effects of SARPE on airway function through hyoid bone movements.

## Introduction

Posterior crossbite is a common malocclusion, affecting about 10% of individuals with permanent dentition globally [1], and up to 15% of adult European populations [2]. It may contribute to breathing disorders associated with transverse maxillary constriction [3].

Rapid palatal expansion (RPE) is a frequent orthodontic intervention for crossbite correction and could have positive effects on hyoid bone position and overall airway morphology [4–6], reducing the risk of obstructive sleep apnea (OSA) [7]. Studies have shown that RPE can lead to repositioning of the hyoid bone, resulting in a more anterior and superior placement relative to its pre-treatment position [8–10].

A more anterior and superior hyoid position is generally associated with improved airway dimensions, potentially reducing the risk of OSA and airway collapsibility during sleep [11].

Conversely, a more inferiorly and posteriorly positioned hyoid bone is often considered a risk factor for OSA, as it may decrease airway space and increase collapse likelihood during sleep [12–14]. Ultrasound studies during drug-induced sleep have shown a connection between increased respiratory effort and caudal hyoid displacement [15, 16]. Although it is unclear whether caudal hyoid displacement is a muscular effect to reduce airway resistance, these studies reveal significant differences between obstructive and non-obstructive breathing conditions.

Questionnaire studies indicate that palatal expansion improves subjective symptoms in children with OSA [17–19]. In theory, RPE expands nasal structures and improves airflow, which is supported by rhinomanometric studies showing increased nasal flow after expansion [19]. While cone-beam computed tomography (CBCT) studies confirm structural widening, its impact on oropharyngeal volume remains inconclusive [20].

However, breathing disorders also affect the vertical pharyngeal airway space (PAS), which includes adjacent structures [21]. Maxillary expansion seems to be associated with a superior and anterior tongue position due to increased palatal space, which could positively influence hyoid position and pharyngeal air passage [21, 22].

The literature presents two main arguments linking maxillary expansion to pharyngeal widening: (1) the potential for a superior-anterior tongue position with cranial hyoid displacement [21], and (2) the improvement of the nasal passage, which may function as a natural positive airway pressure [19].

Since it has been shown that RPE can have a positive effect on hyoid position [10], the question arises if this is also the case for surgically assisted rapid palatal expansion (SARPE). To date, there is a lack of evidence whether SARPE leads to changes in hyoid position that could improve the airway. The present study adds to the literature in this regard.

Despite the recognised potential of SARPE, there is currently no consensus on the surgical procedure [23–25] or selecting between bone-borne and tooth-borne expansion appliances [26]. Theoretically, bone-borne distraction devices should reduce dental side effects and skeletal relapse compared to tooth-borne appliances [27]. However, studies have found no statistically significant differences in skeletal and dental effects or relapse rates between the two appliances [26, 28, 29]. This raises the question whether changes in hyoid position are greater with bone-borne SARPE, which could have beneficial effects on the airway.

Therefore, the aim of this study was to (1) evaluate the differences in hyoid bone position with SARPE, (2) assess whether there are differences with bone-borne or tooth-borne expansion appliances, and (3) investigate possible relationships between expansion values and hyoid bone position. The primary null hypothesis was tested that there is no significant difference in hyoid position before and after SARPE. The secondary null hypothesis was tested that there is no difference in the position of the hyoid bone after SARPE between the bone-borne and tooth-borne group and that hyoid bone position does not change with different amounts of transverse expansion in either the bone-borne or tooth-borne group.

## Methods

This retrospective cohort study received approval from the Local Ethics Committee of the Medical Faculty of the University of Münster, Germany (2021-120-f-S). The study was reported according to the Strengthening the Reporting of Observational Studies in Epidemiology (STROBE) guidelines [30]. Data collection and analysis took place at the Department of Orthodontics, University Hospital Münster, Germany.

Eligible for inclusion in this study were patients who underwent SARPE at the Department of Cranio-Maxillofacial Surgery, University Hospital Münster, Germany from 2010 to 2022. The inclusion criteria were: (1) adult patients over the age of 18, (2) transverse maxillo-mandibular discrepancy manifesting as unilateral or bilateral crossbite affecting the posterior dental segments, (3) Angle Class I, II, or III malocclusion, (4) use of a bone-borne or tooth-borne expansion appliance, (5) lateral cephalograms available at ($t_0$) and ($t_1$). Exclusion criteria were: (1) patients with congenital or developmental anomalies, including craniofacial syndromes, clefts, primary eruption failures and genetic tooth abnormalities, regardless of whether these conditions were accompanied by concomitant systemic diseases, (2) use of a hybrid, i.e. tooth-bone-borne expansion appliance.

**Fig. 1:**
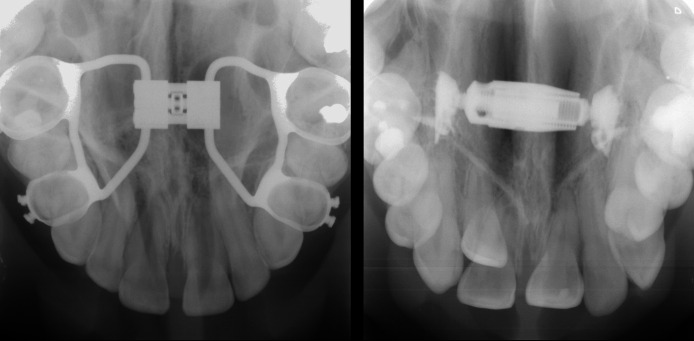
Occlusal radiographs of an individual Hyrax appliance (left) and a standardised transpalatal distractor (right). Radiographs were taken after surgery and before postoperative activation

Two groups were formed to investigate hyoid bone position (Fig. 1): the Hyrax group consisted of patients who underwent SARPE using a tooth-borne expansion appliance consisting of a Hyrax screw (Dentaurum, Ispringen, Germany) soldered to the premolar and molar bands via the retention legs. The TPD group consisted of patients who underwent SARPE using a bone-borne transpalatal distractor (Surgi-Tec, Gent, Belgium) consisting of telescoping titanium cylinders. Both screws were usually placed at the level of the second premolars, with variations depending on the palatal constriction.

A sample size calculation using G*Power 3.1 [31] based on $\alpha = 0.05$ (two-sided) and power of $1 - \beta = 0.80$ was performed. Assuming a clinically meaningful group difference in the vertical hyoid bone position of 2 mm (SD 2.8 mm [32]), an effect size (Cohen’s d) of 0.71 was calculated. These values suggested that each group required a minimum of 32 participants.

All SARPE procedures involved a subtotal Le-Fort I osteotomy with separation of the pterygomaxillary junction. At a later stage, each patient received additional orthognathic surgery for three-dimensional bite correction at the Department of Cranio-Maxillofacial Surgery, University Hospital Münster. Marginal incisions were performed for the surgical procedures as previously described [33]. The decision to use a bone-borne or tooth-borne expansion appliance was made individually by the referring orthodontist; no formal allocation protocol was followed. Intraoperatively, the appliances were activated for 1 mm followed by a latency period of 2 days. Postoperatively, the appliances were activated for 0.5-1 mm per day, depending on the individual situation. After SARPE, a retention period of 6 months was applied before starting orthodontic treatment. To obtain the lateral cephalograms, standard protocols were used. The heads of all patients were aligned with the sagittal plane perpendicular to the X-ray beam and the Frankfurt plane parallel to the floor. The teeth were in maximum intercuspation and the lips closed throughout the exposure.

### Measurement process

All measurements were taken at two time points: before SARPE ($t_0$) and after SARPE ($t_1$). The ($t_1$) cephalograms were obtained prior to any orthognathic surgery, specifically during the preoperative planning for the second surgical procedure.

To determine the amount of maxillary expansion, dental measurements were made on stereolithography (STL) files of the upper jaw (intraoral scan or digitised plaster cast) before treatment and after orthodontic preparation for the second surgical intervention. A detailed description of the transversal measurement methodology was published previously [34]. The following section provides a brief overview of the methodology: STL files of the upper jaw were analysed using Meshmixer (Autodesk, Inc., San Rafael, CA, USA). Pre-treatment STLs were symmetrically aligned in Meshmixer’s world frame and superimposed with the post-treatment STLs. For transverse measurements, a ‘surface object‘ parallel to the median (suture) plane was placed at a specific landmark on one side, then moved to a corresponding landmark on the other side using the transform tool. Arch width was measured between corresponding teeth, including canine tips (C), buccal cusp tips of second premolars (P2), and mesiobuccal cusps of first and second molars (M1, M2) and the amount of maxillary expansion was calculated.

**Fig. 2:**
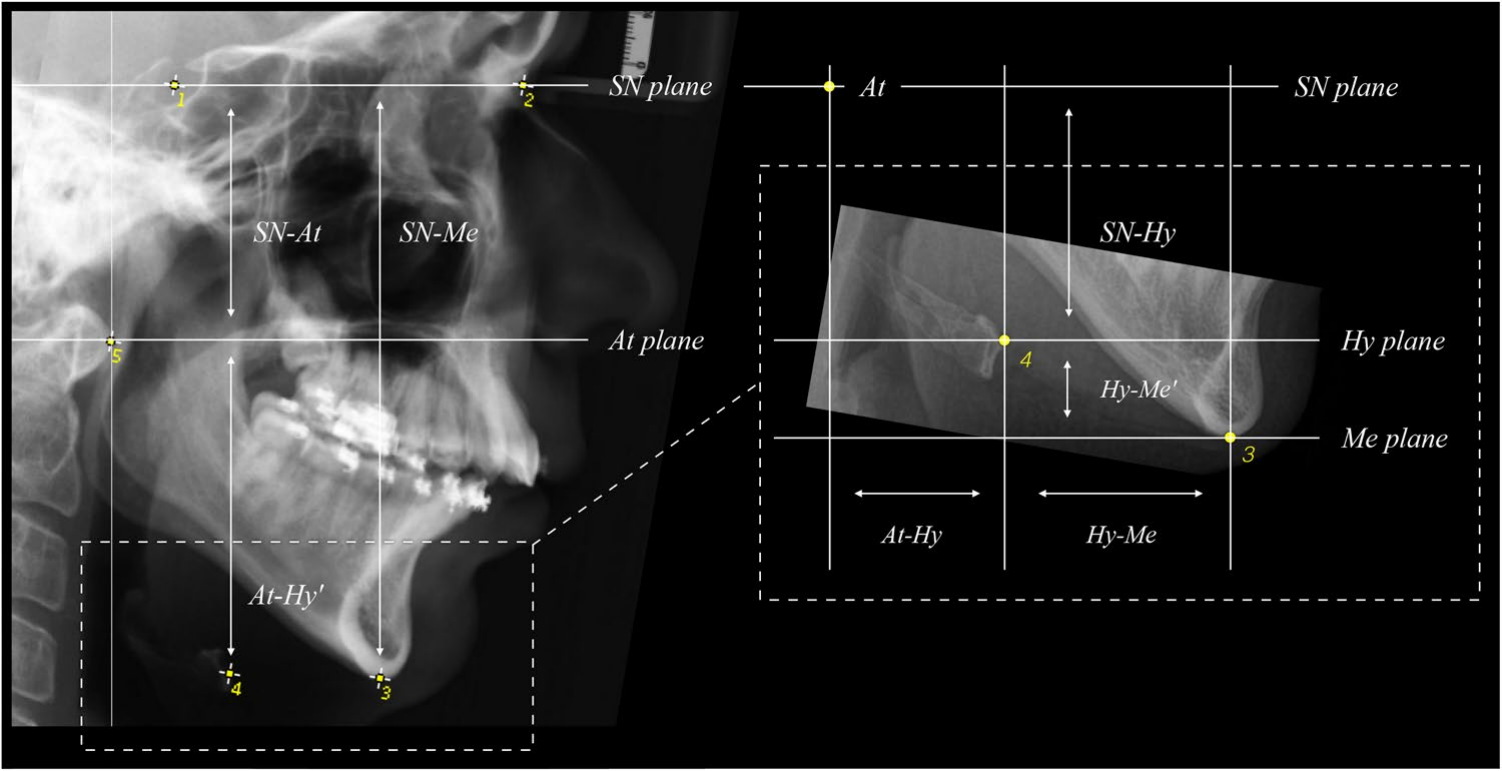
Identified landmarks on the cephalograms: 1=(S)ella: The midpoint of the sella turcica; 2=(N)asion: The most anterior point of the frontonasal suture; 3=(Me)nton: The most inferior point of the mandibular symphysis; 4=(Hy)oid: The uppermost, foremost point on the os hyoideum; 5=(At)las: The foremost point on the Atlas (tuberculum anterius). Constructed planes from the landmarks: SN plane; At plane; Hy plane; Me plane. A description of the calculated distances and measurements is shown in Table 1. For detailed definitions and illustrations of cephalometric landmarks and measurements, please see [36]

To assess hyoid position, lateral cephalograms were analysed at $t_0$ and $t_1$ and differences in hyoid bone position ($t_0$ - $t_1$) were calculated. All landmarks were placed by one experienced investigator (JQS) using ImageJ [35]. To assess the hyoid bone position relative to anatomical structures not affected by SARPE, the following five reliable landmarks were identified: (S)ella, (N)asion, (Me)nton, (Hy)oid, and (At)las (Fig. 2). The Sella-Nasion plane (SN plane) was used as a reference plane, while the Atlas plane (At plane), Hyoid plane (Hy plane), and Menton plane (Me plane) were oriented parallel to it. A script was developed to compute the following measurements from the landmark coordinates: the vertical distances SN-Me, SN-At, At-Hy’, Hy-Me’ (where ’ indicates vertical distances) and the sagittal distances At-Hy, Hy-Me (Fig. 2, Table 1). To evaluate the changes in craniocervical inclination between pre- and postoperative lateral cephalograms, the angle $\alpha$ formed by the intersection of the Atlas-Sella and Sella-Nasion lines at Sella point was computed.

**Table 1:** Description and intraclass correlation coefficients (ICC) of the calculated distances and measurements

Measurement	Description	ICC
$\alpha$ [$^{\circ }$]	Angle formed by the intersection of the Atlas-Sella and Sella-Nasion lines at Sella point	0.811
SN-Me [mm]	Vertical distance from the Sella-Nasion plane to the Menton point, measured perpendicular to the Sella-Nasion plane	0.883
SN-At [mm]	Vertical distance from the Sella-Nasion plane to the Atlas point, measured perpendicular to the Sella-Nasion plane	0.973
SN-Hy [mm]	Vertical distance from the Sella-Nasion plane to the Hyoid point, measured perpendicular to the Sella-Nasion plane	0.976
At-Hy’ [mm]	Vertical distance from the Atlas plane to the Hyoid plane, measured perpendicular to the Sella-Nasion plane	0.941
At-Hy [mm]	Sagittal distance from the Atlas point to the Hyoid point measured parallel to the Sella-Nasion plane	0.982
Hy-Me [mm]	Sagittal distance from the Hyoid point to the Menton point measured parallel to the Sella-Nasion plane	0.990
Hy-Me’ [mm]	Vertical distance from the Hyoid point to the Menton point, measured perpendicular to the Sella-Nasion plane; positive values indicate that Hyoid point is cranial of Menton point	0.947

#### Calculation of distances

The calculation of the distances is based on the concept of vector projection and rejection as defined below.

$$\begin{aligned} \begin{array}{ll} \text {Vector projection:} \qquad \qquad & \qquad \qquad \\ & \qquad \qquad \qquad \text {Vector rejection:} \\ \qquad \qquad \vec {a}_{\parallel \vec {b}} = \frac{\vec {a}\cdot \vec {b}}{\vec {b}\cdot \vec {b}}\vec {b} \qquad & \qquad \qquad \qquad \qquad \qquad \vec {a}_{\perp \vec {b}} = \vec {a} - \vec {a}_{\parallel \vec {b}} \end{array} \end{aligned}$$

$$\begin{aligned} \begin{array}{ll} & \text {Vertical measurements:} \\ \text {Sagittal measurements:} & \\ \text{At-Hy} = \left\| \vec {At}_{\parallel (\vec {Na}-\vec {S})} - \vec {Hy}_{\parallel (\vec {Na}-\vec {S})} \right\| & \qquad \,\,\, \text {SN-At} = \left\| (\vec {At} - \vec {S})_{\perp (\vec {Na}-\vec {S})} \right\| \\ & \qquad \,\,\text {SN-Me} = \left\| (\vec {Me} - \vec {S})_{\perp (\vec {Na}-\vec {S})} \right\| \\ \text {At-Me} = \left\| \vec {At}_{\parallel (\vec {Na}-\vec {S})} - \vec {Me}_{\parallel (\vec {Na}-\vec {S})} \right\| & \qquad \,\,\,\text {SN-Hy} = \left\| (\vec {Hy} - \vec {S})_{\perp (\vec {Na}-\vec {S})} \right\| \\ & \qquad \,\,\,\,\,\, \text {At-Hy'} = \text {SN-Hy} - \text {SN-At} \\ \text {Hy-Me} = \text {At-Me} - \text {At-Hy} & \qquad \,\,\,\,\, \text {Hy-Me'} = \text {SN-Me} - \text {SN-Hy} \end{array} \end{aligned}$$

### Statistical analysis

To assess the normality of the data distribution, the Shapiro-Wilk test were used, which indicated that the data were not normally distributed ($p<0.05$). Given the sample sizes, descriptive statistics were calculated for all variables and were reported as means and standard deviations (SD) to describe the central tendency and variability of the data. Chi-squared tests and Mann-Whitney U tests were used to evaluate differences in the baseline characteristics. Wilcoxon signed-rank tests were used to investigate intragroup differences and Mann-Whitney U tests were used to evaluate intergroup differences. Pearson correlation coefficients were calculated to assess possible relationships between expansion values and hyoid bone position. The significance level was set to $\alpha = 5\%$. No alpha correction was performed due to the exploratory nature of the study. Intrarater reliability for the cephalometric measurements was evaluated using intraclass correlation coefficients (ICC). For this purpose, 10% of the cases were randomly selected using a random number generator [37] and re-evaluated by the principal investigator after at least 4 weeks. ICC estimates were calculated based on a single measurement, absolute-agreement, 2-way mixed effects model. Interpretation of the correlation coefficients followed the cut-off limits of [38]. SPSS Statistics 29 (IBM Corp., Armonk, NY, USA) was used to perform all statistical analyses.

## Results

A total of 65 cases met the inclusion criteria out of 91 patients who received SARPE during the study period. The baseline characteristics are presented in Table 2. In both groups, the age distributions for females and males were relatively similar, with no statistically significant differences. The mean age at $t_0$ in the Hyrax group was 26.0 ± 9.1 years and 25.0 ± 8.0 years in the TPD group. All Angle Classes occurred in both groups, with Class III being the most common. Overall, there were no significant differences in the baseline characteristics between the two intervention groups.

**Table 2:** Hyrax and TPD group baseline characteristics

	Hyrax	TPD	*p*
Age $t_0$ (years) Mean ± SD	26.0 ± 9.1	25.0 ± 8.0	0.613
Age $t_1$ (years) Mean ± SD	28.8 ± 9.0	27.7 ± 7.6	0.669
Gender n (%)			0.168
Female	20 (62.5%)	15 (45.5%)	
Male	12 (37.5%)	18 (54.5%)	
Angle Class n (%)			0.395
Class I	1 (3.1%)	4 (12.1%)	
Class II	11 (34.4%)	10 (30.3%)	
Class III	20 (62.5%)	19 (57.6%)	

Intrarater reliability was excellent for all variables except for angle $\alpha$ and SN-Me, which showed good reliability (Table 1). Sagittal measurements tended to exhibit slightly higher reliability than vertical measurements, with the Hy-Me distance showing the highest reliability. Mean expansion values with SARPE for both groups are shown in Table 3. Expansion values for all measurement sites were higher in the TPD group compared to the Hyrax group. However, this difference was not statistically significant ($p>0.05$).

**Table 3:** Descriptive statistics for SARPE expansion values in millimeters ($t_1$ - $t_0$) and intergroup Mann-Whitney U test statistics

	Hyrax				TPD				
	Mean	SD	Min.	Max.	Mean	SD	Min.	Max.	*p*
C	2.24	1.92	-0.85	5.22	3.08	3.91	-1.96	11.11	0.577
P2	5.53	2.08	1.37	9.25	7.06	2.47	3.07	10.89	0.166
M1	5.03	2.14	1.42	8.40	7.00	3.12	2.21	14.79	0.103
M2	4.25	2.13	0.27	7.91	5.79	3.30	2.34	11.13	0.443

Measurements were taken at canines (C), second premolars (P2), first molars (M1), and second molars (M2)

Table 4 shows the combined results for the hyoid bone position in the Hyrax and TPD groups before ($t_0$) and after SARPE ($t_1$), and Table 5 shows the differences ($t_0$ - $t_1$) for the hyoid bone position. In general, hyoid bone position remained stable after SARPE. Notably, only the Hy-Me distance decreased significantly from 44.23 mm to 43.40 mm ($p=0.005$) indicating that the hyoid moved 1.79 mm more anteriorly ($\Delta$ Hy-Me) after SARPE. All other measurements showed no significant changes.

**Table 4:** Descriptives and Wilcoxon signed-rank test statistics before ($t_0$) and after SARPE ($t_1$) for both groups

	SARPE (Hyrax + TPD) ($t_0$)				SARPE (Hyrax + TPD) ($t_1$)				
	Mean	SD	Min	Max	Mean	SD	Min	Max	*p*
$\alpha$	110.06	7.30	91.54	127.05	109.94	6.79	89.80	126.31	0.080
SN-Me	115.04	9.04	93.73	139.16	116.46	9.89	91.69	144.07	0.243
SN-At	45.49	5.34	35.30	60.73	46.04	5.56	35.20	62.57	0.352
SN-Hy	106.50	9.24	90.96	125.09	106.21	10.26	86.26	126.89	0.883
At-Hy’	61.01	6.69	48.62	77.89	60.17	8.27	45.61	83.38	0.669
At-Hy	15.20	8.62	1.29	35.33	16.60	8.50	0.61	38.31	0.091
Hy-Me	44.23	7.96	28.06	60.15	43.40	6.67	29.26	60.27	0.005*
Hy-Me’	8.43	7.29	-5.82	24.90	10.25	7.87	-9.58	29.59	0.291

* p <0.05

**Table 5:** Descriptive statistics for differences in hyoid bone position ($t_0$ - $t_1$) with SARPE (Hyrax + TPD)

	SARPE (Hyrax + TPD)			
	Mean	SD	Min	Max
$\Delta$ $\alpha$	0.55	2.85	-8.13	13.67
$\Delta$SN-Me	-0.66	4.17	-14.89	7.83
$\Delta$SN-At	-0.67	3.40	-17.40	4.26
$\Delta$SN-Hy	-0.21	5.22	-15.59	10.56
$\Delta$At-Hy’	0.46	4.83	-8.99	12.11
$\Delta$At-Hy	-0.89	3.63	-11.36	6.24
$\Delta$Hy-Me	1.79	4.72	-8.29	14.12
$\Delta$Hy-Me’	-0.60	3.37	-8.60	6.12

Negative values mean greater$t_1$values

### Intragroup differences

In the Hyrax group, hyoid bone position generally remained stable after SARPE (Table 6). There was a statistically significant difference between $t_0$ and $t_1$ only for Hy-Me and Hy-Me’. The sagittal distance Hy-Me decreased from 44.23 mm to 41.80 mm ($p=0.002$), and the vertical distance Hy-Me’ increased from 8.43 mm to 10.38 mm ($p=0.004$) indicating that the hyoid moved anteriorly and superiorly in the Hyrax group. All other measurements showed no significant changes.

**Table 6:** Hyrax group descriptives and Wilcoxon signed-rank test statistics before ($t_0$) and after SARPE ($t_1$)

	Hyrax ($t_0$)				Hyrax ($t_1$)				
	Mean	SD	Min	Max	Mean	SD	Min	Max	*p*
$\alpha$	110.06	7.30	91.54	127.05	110.08	7.52	89.80	126.31	0.993
SN-Me	115.04	9.04	93.73	139.16	116.10	9.14	96.07	144.07	0.331
SN-At	45.49	5.34	35.30	60.73	45.51	5.61	35.20	59.11	0.779
SN-Hy	106.50	9.24	90.96	125.09	105.74	10.32	86.26	126.89	0.210
At-Hy’	61.01	6.69	48.62	77.89	60.22	7.47	45.80	75.40	0.379
At-Hy	15.20	8.62	1.29	35.33	15.90	9.07	1.95	38.31	0.161
Hy-Me	44.23	7.96	28.06	60.15	41.80	6.63	29.26	55.13	0.002*
Hy-Me’	8.43	7.29	-5.82	24.90	10.38	7.56	-2.97	29.59	0.004*

* p <0.05

Table 7 presents the descriptive statistics and Wilcoxon signed-rank test results for the TPD group. Hyoid bone position also remained stable after SARPE. This was also the case for Hy-Me and Hy-Me’, which showed no significant changes. Only the $\alpha$ angle showed a statistically significant difference, decreasing from 110.90$^{\circ }$ at ($t_0$) to 109.81$^{\circ }$ at ($t_1$) ($p=0.037$).

**Table 7:** TPD group descriptives and Wilcoxon signed-rank test statistics before ($t_0$) and after SARPE ($t_1$)

	TPD ($t_0$)				TPD ($t_1$)				
	Mean	SD	Min	Max	Mean	SD	Min	Max	*p*
$\alpha$	110.90	5.34	101.90	124.26	109.81	6.11	95.21	124.56	0.037*
SN-Me	116.52	10.96	93.20	133.95	116.80	10.70	91.69	134.77	0.526
SN-At	45.24	3.88	38.02	52.71	46.54	5.56	36.16	62.57	0.114
SN-Hy	105.51	10.18	85.77	123.50	106.67	10.34	91.06	126.57	0.122
At-Hy’	60.27	9.29	43.54	78.70	60.13	9.10	45.61	83.38	0.623
At-Hy	16.20	7.67	1.25	32.92	17.27	7.98	0.61	37.42	0.339
Hy-Me	46.19	8.15	28.69	62.17	45.01	6.42	31.98	60.27	0.286
Hy-Me’	10.89	8.50	-9.12	23.60	10.11	8.28	-9.58	24.38	0.155

* p < 0.05

### Intergroup differences

Table 8 summarises the changes in hyoid bone position ($t_0$ - $t_1$) for the Hyrax and TPD groups, along with intergroup test statistics.

Statistically significant findings were observed only in the variables angle $\alpha$ and Hy-Me’. The $\alpha$ angle decreased slightly in the Hyrax group by 0.02° while increasing in the TPD group by 1.25°, resulting in a significant intergroup difference ($p=0.036$). The vertical distance Hy-Me’ significantly decreased in the Hyrax group (mean difference -1.95 mm) while increasing in the TPD group (mean difference 0.75 mm) ($p=0.001$), indicating that the hyoid moved superiorly in the Hyrax group and inferiorly in the TPD group.

The change in the vertical distance SN-Hy showed an increase in the Hyrax group by 0.77 mm and a decrease in the TPD group by -1.48 mm. However, this intergroup difference was not statistically significant ($p=0.070$).

All remaining variables showed no statistically significant differences between the two groups.

**Table 8:** Descriptive statistics for differences in hyoid bone position ($t_0$ - $t_1$) and intergroup Mann-Whitney U test statistics

	Hyrax				TPD				
diff	Mean	SD	Min.	Max.	Mean	SD	Min.	Max.	*p*
$$\Delta$$$$\alpha$$	-0.02	1.45	-3.14	3.52	1.25	3.64	-8.13	13.67	0.036*
$\Delta$SN-Me	-1.06	4.64	-14.89	7.83	-0.47	3.56	-6.04	7.32	0.788
$\Delta$SN-At	-0.02	2.28	-6.60	4.05	-1.31	4.22	-17.40	4.26	0.185
$\Delta$SN-Hy	0.77	5.96	-15.59	10.56	-1.48	3.93	-8.32	5.34	0.070
$\Delta$At-Hy’	0.79	4.91	-8.99	8.62	-0.16	4.54	-6.45	12.11	0.431
$\Delta$At-Hy	-0.70	2.89	-8.47	4.75	-1.10	4.34	-11.36	6.24	0.723
$\Delta$Hy-Me	2.43	4.28	-7.71	14.12	1.14	5.11	-8.29	10.92	0.301
$\Delta$Hy-Me’	-1.95	3.19	-8.60	3.87	0.75	3.03	-6.02	6.12	0.001*

Negative values mean greater$t_1$values

* p <0.05

### Correlation analysis

The correlation analysis examined the relationship between cephalometric variables of hyoid bone position and the amount of expansion in both Hyrax and TPD groups. Overall, correlations between the expansion values and the position of the hyoid bone were inconclusive.

In the Hyrax group, a negative correlation was identified between canine expansion and the distance Hy-Me (Pearson: -0.522; $p = 0.038$), indicating that as expansion increases, the hyoid moved anteriorly. No significant correlations were found for other expansion measures in this group.

In the TPD group, expansion at P2 correlated positively with craniocervical inclination (Pearson: 0.696; $p = 0.017$), while expansion at M2 correlated negatively with Hy-Me (Pearson: -0.765; $p = 0.010$), indicating a decrease in Hy-Me distance and anterior movement of the hyoid with increased expansion.

**Fig. 3:**
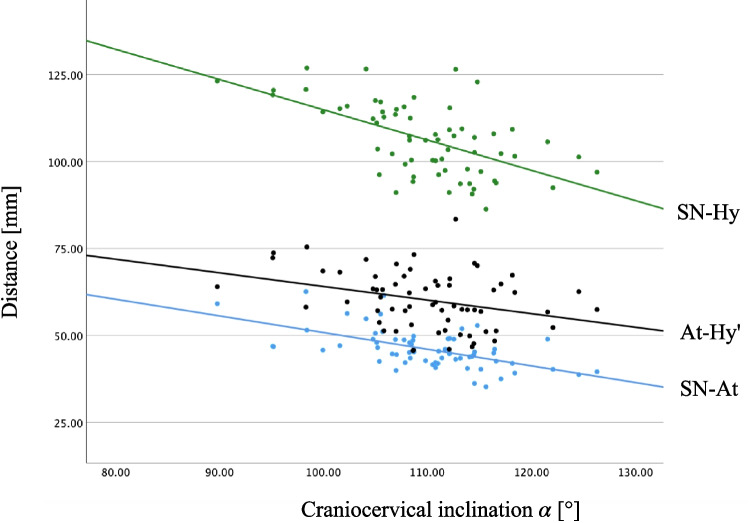
Scatter plot of the cephalometric distance SN-Hy, At-Hy’ and SN-At in relation to the craniocervical inclination formed by the angle between SN and At

Across both groups, craniocervical inclination ($\alpha$) was correlated with the distances SN-At, At-Hy’, and SN-Hy (Pearson values range from -0.321 to -0.585, all significant) as shown in Fig. 3 even though these distances remained stable within their respective groups.

## Discussion

This study aimed to investigate the effects of SARPE on hyoid bone position and to compare the outcomes between bone-borne and tooth-borne expansion appliances. While the null hypotheses were rejected, we found that hyoid bone position remained generally stable post-treatment with no changes exceeding the threshold of 2 mm, which was considered clinically significant [10]. Statistically significant changes were observed only for the Hy-Me distance, which demonstrated a slight anterior movement of the hyoid bone after SARPE in the overall cohort. Regarding bone-borne and tooth-borne SARPE, there was only a statistically significant difference for Hy-Me’ and $\alpha$. Hy-Me’ decreased with Hyrax while increasing with TPD, indicating that the hyoid moved superiorly in the Hyrax group and inferiorly in the TPD group. Correlations between the expansion values and the position of the hyoid bone were inconclusive.

One of the key findings of this study was the small anterior displacement of the hyoid bone. This is consistent with previous literature suggesting that maxillary expansion can positively influence hyoid bone position [10]. The anterior positioning of the hyoid bone might be associated with an increased airway space, potentially contributing to improved respiratory function and reduced OSA risk [11]. However, while our results indicate some anterior displacement, the overall changes in hyoid bone position were not substantial enough to draw definitive conclusions regarding the clinical impact on obstructive breathing disorders. When interpreting the results it seems important to consider that in both groups the expansion had an effect on the sagittal decrease of Hy-Me. This could be the result of an anterior displacement of the hyoid or a posterior movement of the chin. Since the sagittal distance from the hyoid to the spine (At-Hy) did not show any significant changes, it must be assumed that this distance is stable, which ultimately favors a posterior movement of the chin. We assume that this is due to a small autorotation of the mandible after caudal displacement of the mobilised maxilla with expansion. However, as our study did not include additional skeletal angular measurements such as SNB or mandibular plane angle, we are unable to directly confirm mandibular positional changes.

There was also a significant difference in the vertical distance Hy-Me’ between the groups, indicating that the hyoid moved superiorly in the Hyrax group and inferiorly in the TPD group. Again, the question is whether there was a superior movement of the hyoid or an inferior movement of the chin. Considering both groups, the craniocervical inclination $\alpha$ correlated with the distances SN-At, At-Hy’, and SN-Hy. Since each of these parameters did not differ within its group, the distances SN-At, SN-Hy and At-Hy’ must be seen as stable but move with the craniocervical inclination. Therefore, the vertical change of Hy-Me’ cannot be regarded as an isolated movement of the hyoid. The maximum expansion values of the SARPE in both groups (up to 14 mm) represent a significant intervention. If there were a causal relationship, this should have been reflected in a superior movement of the hyoid. The data suggest a clockwise rotation of the mandible and therefore a clockwise rotation of Me, especially in the Hyrax group. It can be speculated that the tooth-borne appliance causes a bite opening that is still present after orthodontic preparation for the final surgery.

In summary, the overall changes in hyoid bone position were not substantial enough to draw definitive conclusions regarding the clinical impact on the airway. These findings must be interpreted with caution. The significant variability within the sample and the relative movements of the hyoid bone alongside other craniofacial structures could potentially mask or misinterpret the true effects of SARPE. Although some measurements of our study were statistically significant, a clinical effect is rather doubtful. Hyoid bone changes did not exceed the threshold of 2 mm, which was considered clinically significant. We cannot interpret this small distance as a clear effect on tongue position or on the retropharyngeal space, especially since in the TPD group expansion values were greater and hyoid position remained stable.

Since the hyoid bone is attached to the mandible, tongue, styloid processes, thyroid cartilage, and cricoid cartilage, it is reasonable to assume that a therapeutically induced change in hyoid position will affect deeper airway structures. Maxillary expansion is considered a possible treatment to elevate the tongue and the associated hyoid bone by increasing palatal volume [4, 5, 7]. Cephalometric studies of tongue position using radiopaque solutions are limited in the literature [39, 40], so tongue position is mainly assessed indirectly via voice function [41]. A possible link between maxillary expansion and oropharyngeal expansion via the hyoid and tongue is also seen in the positive reports of patients who experienced improved nasal flow after expansion [19]. Despite a measurable increase in nasal volume, the respiratory effect is controversial [20, 41] and SARPE may not be understood as a promise of healing breathing disorders [42]. To date, there is a lack of evidence whether SARPE leads to changes in hyoid position that could improve the airway. The present study adds to the literature in this regard. Our results do not confirm this evidence. The hypothesis that the position of the hyoid bone changes depending on the amount of maxillary expansion could not be confirmed and the hypothesis that changes in hyoid position are greater with bone-borne SARPE must also be rejected.

This assumption of superior movement of the hyoid relative to the mandible was confirmed by Phoenix and coworkers [10]. However, [43] concluded that the observed changes in hyoid and tongue position were mainly related to growth rather than the RPE intervention itself.

Our results are in line with previous findings that found stable sagittal hyoid positions and inconclusive vertical positions in the same distance range [4, 9, 10, 44–46]. In the majority of studies, superior displacement of the hyoid is considered to be biased by posterior rotation of the mandible [9].

### Strengths and limitations

This study has several strengths that make it a valuable addition to the literature. First, the cohort design allowed for the inclusion of a reasonable sample size of 65 patients who underwent SARPE, ensuring that the results were derived from diverse cases reflecting real-world clinical scenarios. All patients received a similar surgical protocol, which strengthens the validity of the study by reducing confounding variables. Additionally, established methods were employed to assess hyoid bone position, resulting in high intraclass correlation coefficients that underline the reliability of the data.

Despite these strengths, this study has several limitations that need to be considered when interpreting the results. The retrospective design introduces potential selection bias, which may affect generalisability. Additionally, as the choice of appliance (bone-borne or tooth-borne) was made individually by the referring orthodontist rather than by formal allocation, there is a possibility of selection bias between the study groups, which may affect the interpretability of the group comparisons. While cephalometric analysis is a common method for assessing hyoid position, its precision may have limitations [47]. Taking lateral cephalograms at different stages of respiration (inhalation/ exhalation) and swallowing cycles could also affect the measurements of hyoid bone position [48, 49]. However, recent research supports the reliability of these measurements [50, 51]. Although standard protocols were used during lateral cephalogram acquisition, minor variations in natural head posture may have persisted. Such variations can affect vertical and angular cephalometric values, including the craniocervical angle ($\alpha$), and thus represent a limitation of this study.

CBCT studies on orthognathic surgery, malocclusion, and growth patterns are numerous. However, those specifically examining the hyoid bone before and after SARPE are limited. Existing studies generate cephalometric images from 3D data for sagittal measurements [52], and these studies also indicate that there are no differences in either tongue [53] or hyoid position following SARPE [44, 53]. The inherent variability in individual responses to treatment means that our results should be interpreted cautiously, particularly when considering the clinical significance of the findings. The influence of craniocervical inclination is also debated, as the differences reported in the literature do not represent clinically relevant measures [19] and are more dependent on the device used than on the expansion itself [54]. Another limitation of this study is the absence of a control group. The lack of such comparators limits our ability to detect the effects of SARPE more definitively. A further limitation of this study is that no direct measurements of respiratory function were obtained. Therefore, any hypothesised connection between changes in hyoid bone position and airway function remains speculative in the absence of objective functional data.

Future research including a control group could benefit from a longitudinal design that includes multiple follow-up assessments to better evaluate the long-term changes in hyoid position and airway after SARPE. Direct respiratory assessments are needed to investigate the impact of hyoid position changes on airway function.

## Conclusion

While the results indicate small, statistically significant, but probably clinically negligible movements of the hyoid bone after SARPE, the hyoid position remained generally stable and changes were not dependent on the appliance used or the amount of expansion. Overall, the observed changes were not substantial enough to support any clinically meaningful effects of SARPE on airway function and obstructive sleep apnea management through hyoid bone movements.

## Data Availability

The data presented in this study are available on reasonable request from the corresponding author.
